# Agenesis of the internal carotid artery

**DOI:** 10.1590/1677-5449.001918

**Published:** 2018

**Authors:** Adriano Carvalho Guimarães, Thaís Duarte Baião Pessoa, Ricardo Herkenhoff Moreira, Walter Junior Boim de Araujo

**Affiliations:** 1 V&P Health Excelência Médica, Santo Antônio da Platina, PR, Brasil.; 2 Hospital Angelina Caron, Serviço de Cirurgia Vascular, Campina Grande do Sul, PR, Brasil.; 3 Hospital Nossa Senhora da Saúde, Santo Antônio da Platina, PR, Brasil.; 4 Universidade Federal do Paraná (UFPR), Departamento de Cirurgia, Curitiba, PR, Brasil.

**Keywords:** carotid artery, agenesis, intracranial aneurysm

## Abstract

Agenesis of the internal carotid artery is a rare anomaly. It is usually asymptomatic because of the presence of anastomoses, but it can be associated with complications, especially when there is evidence of other anatomical abnormalities or severe atherosclerotic disease. We report the case of a 63-year-old female patient with hypertension and diabetes and a history of intracranial aneurysm clipping. Doppler ultrasonography and computed tomography angiography of the carotid and vertebral arteries showed unilateral agenesis of the left internal carotid artery. This report aims to highlight the importance of suspecting vascular malformations during investigation of neurological conditions. Internal carotid agenesis has a significant association with intracranial aneurysms and their early detection can spare the patient serious complications.

## INTRODUCTION

 The cervical and cerebral arterial system undergoes many transformations during the process of embryonic development before arriving at its final form in the fetus. Development of this system is modulated by countless molecular factors and failures in these pathways can cause anatomic variants and a range of different clinical repercussions. The primitive aorta has six arches that are organized into the different branches known. The third arch gives rise to the common carotids and the proximal segments of the internal carotids. The distal segments are derived from the dorsal aorta between the first and third primitive arches. The external carotids emerge from the common carotids. This development pattern occurs in approximately 65% of the population; anomalies are observed in the remainder. 1

 Anomalies result from abnormal persistence or disappearance of segments of the arch of the primitive aorta. In 22% of the population, the left common carotid artery originates from the brachiocephalic trunk, rather than the aortic arch, also known as the “bovine aortic arch”. In this case, the brachiocephalic trunk gives rise to the right subclavian artery and the left and right common carotid arteries, while the left subclavian artery originates from the aortic arch, as normally expected. This variant accounts for 73% of all anomalies of the arch. Many other variants have been described, all of which occur in less than 3% of the population. 

 Agenesis of the internal carotid was described for the first time in 1787, post-mortem, and in vivo for the first time in 1954, after an angiography examination. 2 This is a rare anomaly with incidence of less than 0.01% 3 and, in the majority of cases, it is asymptomatic because anastomoses are present. However, it can be linked with complications, primarily when other anatomic abnormalities or severe atherosclerotic disease are present. 

## CASE REPORT

**Figure 1:**
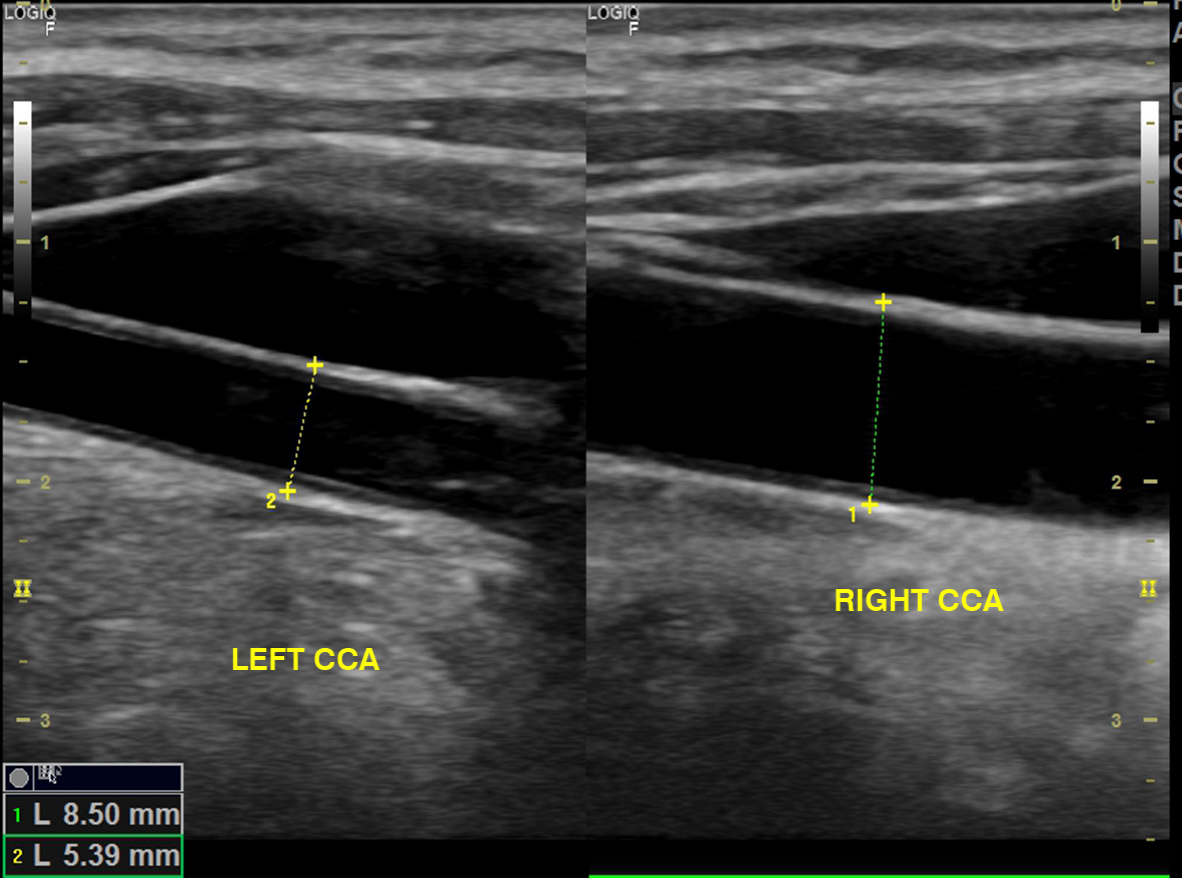
Mode B ultrasonography images showing the left common carotid artery () with a smaller caliber than the right common carotid artery (RIGHT CCA).

**Figure 2:**
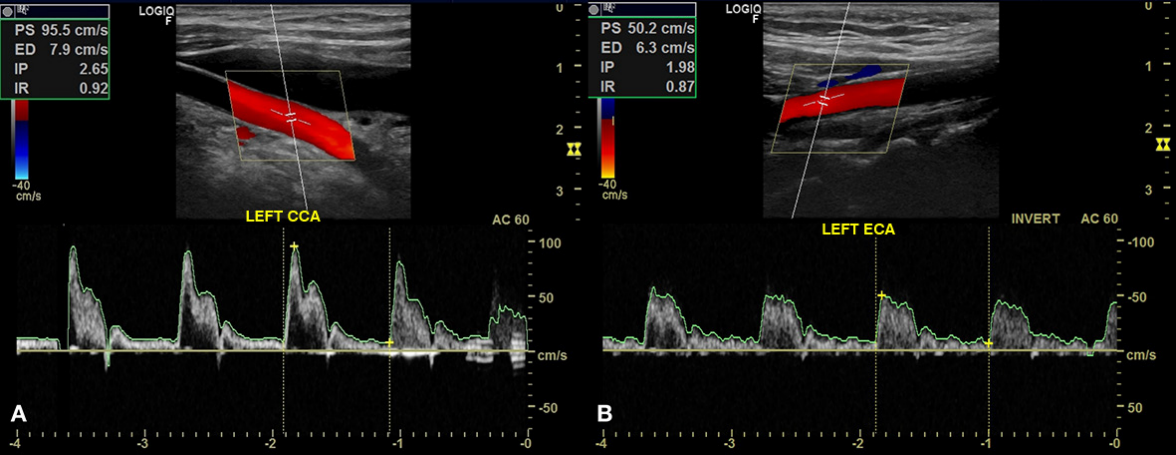
Doppler ultrasonography images showing a patent left common carotid artery (LEFT CCA) (A) leading to the left external carotid artery (LEFT ECA) (B).

**Figure 3:**
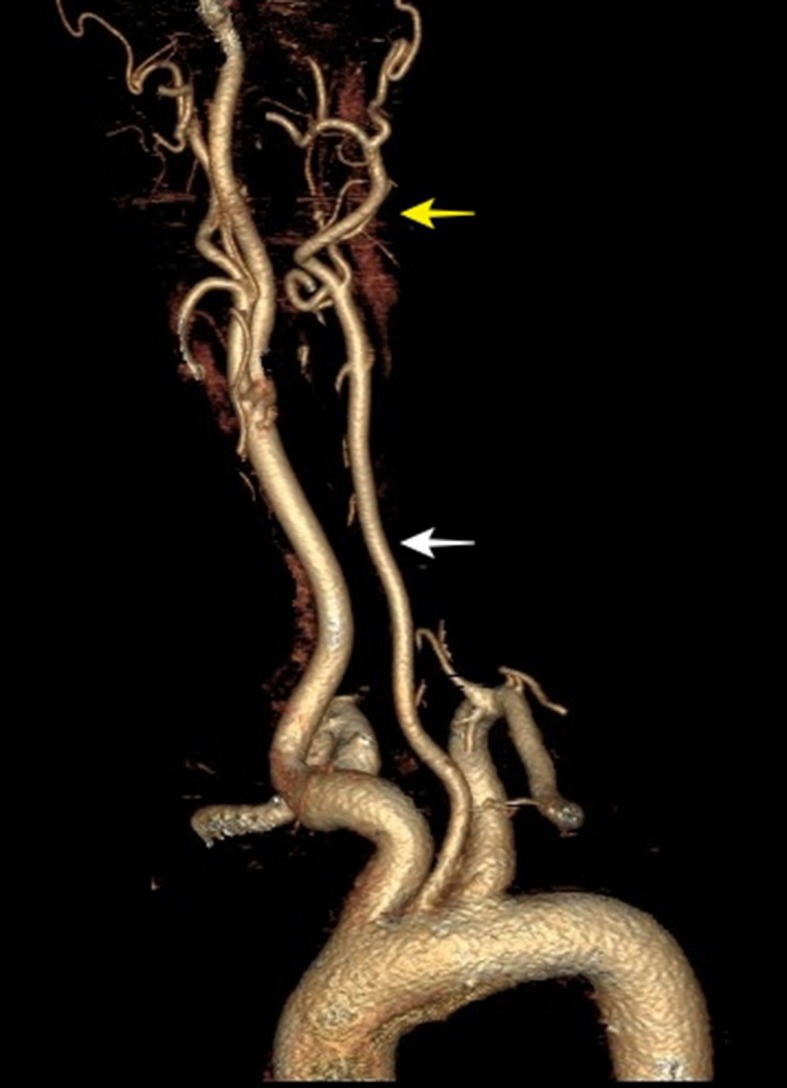
Angiotomography showing a patent left common carotid artery (LEFT CCA) (white arrow) leading to the left external carotid artery (LEFT ECA) (yellow arrow) and agenesis of the left internal carotid artery (LEFT ICA).

 The patient was a 63-year-old female with hypertension and diabetes. She had no history of smoking or heart disease. She had undergone surgery to clip a cerebral aneurysm 3 years previously and the treating neurosurgeon responsible at the time had reported difficulty with catheterization of cervical arteries. She was examined with Doppler ultrasonography of carotid and vertebral arteries, which showed that the left common carotid artery had a smaller caliber than the right ( Figure 1 ), the left carotid bifurcation could not be observed, and the left common carotid artery only led to the left external carotid artery ( Figure 2 ). Angiotomography was ordered, showing agenesis of the left internal carotid artery ( Figure 3 ). The patient remains asymptomatic and attends regular follow-up consultations. 

## DISCUSSION

 Agenesis of the internal carotid is generally unilateral. In these cases, the principal blood supply compensating for the absence is the contralateral internal carotid. In cases with bilateral agenesis, the vertebrobasilar system may fulfill this function. The majority of cases do not exhibit symptoms, which can be attributed to an abundant network of anastomoses, including the circle of Willis, intercavernous vessels, branches of the external carotid, and also remaining embryological arteries. A slight predominance among men and a preference for the left, at a ratio of 3:1, have been reported. 4 ^-^ 6

 According to Lie, the term agenesis describes a complete absence of an organ or structure, whereas aplasia is caused by a lapse in development of an organ – the precursor of which is present – and hypoplasia is the term attributed to incomplete development. It is believed that unilateral agenesis is caused by mechanical or hemodynamic intrauterine stress. The principal hypothesis is that there is an excessive rotation of the embryo in one direction or constriction by an amniotic band. The cause of bilateral agenesis is still unknown. 5 ^,^ 7

 Development of the internal carotid initiates at a point at which the embryo has a 4-5 mm crown-rump length (CRL: the distance from the top of the cranium to the mid-point between the apexes of the buttocks) and is completed during the 6th week of gestation (CRL =10-14 mm). The circle of Willis forms at the 7-24 mm stage (gestational age of around 7 weeks). The collateral blood flow pattern, if there is a carotid malformation, and the intracranial vasculature depend on the stage at which development of the artery is interrupted. Cali et al. postulated that if development of the internal carotid is interrupted before conclusion of the circle of Willis, then collateral circulation will prevail through the primitive vessels (intercavernous anastomoses). If the interruption occurs after the circle is concluded, then collateral circulation will predominantly flow through this route. Few cases of internal carotid agenesis have been described in children, suggesting that collateral vessels are initially able to compensate for the absent flow. The majority of symptomatic cases are identified in adults, among whom factors such as atherosclerosis may precipitate cerebrovascular insufficiency. 2 ^,^ 8

 Lie described six collateral circulation patterns associated with absence of internal carotids. In type A, there is a unilaterally absent internal carotid and collateral circulation through the ipsilateral anterior cerebral artery (ACA) via the anterior communicating artery (ACOM) and the ipsilateral middle cerebral artery (MCA) via the posterior communicating artery (PCOM), which is usually hypertrophic. In type B, there is a unilaterally absent internal carotid combined with collateral circulation through the ACA and MCA via the ACOM. In type C, agenesis is bilateral and anterior circulation is supplied via the vertebrobasilar system through a hypertrophic PCOM. Type D is characterized by unilateral agenesis of the cervical portion of the internal carotid and intercavernous collaterals to the intercavernous segment of the contralateral internal carotid. In type E, there is bilateral hypoplasia of the internal carotids and their ACA and MCA are supplied by hypertrophic PCOMs. In type F, there is bilateral absence which is compensated for by transcranial anastomoses and branches of the carotid via a *rete mirabile*. 7 Since Lie published these descriptions in 1968, other authors have reported presence of a persistent trigeminal artery surrounded by collaterals supplying flow in a patient with unilateral agenesis of the internal carotid. 5 It is important to identify compensatory anastomoses – such as intercavernous anastomoses – to avoid devastating perioperative complications in procedures such as trans-sphenoid access to the pituitary. 

 Other anomalies, such as intracranial aneurysms, may be present in around 24-67% of agenesis cases and can be associated with intracranial hemorrhages, whereas aneurysms are only found in 2-4% of the normal population. Around 25% of symptomatic cases of agenesis can progress to aneurysms, with bleeding. The cause of this elevated prevalence may lie in the fact that there is increased flow through collateral vessels, increasing the stress on their walls. It has been reported that the ACA is the collateral most often involved by aneurysms in this situation. Some authors describe subarachnoid bleeding secondary to aneurysms as the initial finding in cases of agenesis of the internal carotid. 6 In the case described here, there was an aneurysm that had been treated before it caused complications. Some patients may exhibit delayed neuropsychomotor development, headaches, and transitory ischemic episodes. 3 ^,^ 4

 Since this anomaly is asymptomatic in the majority of cases, it is generally diagnosed as an incidental finding in imaging exams. When this occurs, it is important to rule out the possibility that the hypoflow identified is caused by an acquired stenosis or a congenital anomaly of the vessel or bony canal. 3 ^,^ 4 The importance of this differentiation lies in the possibility that the agenesis could be associated with other anomalies, such as aneurysms, which should be identified before complications occur. 

 The anomaly may be detected in a Doppler ultrasonography examination in which it is not possible to observe the carotid bifurcation or in which hypoplasia of the ipsilateral common carotid is visible, as in the case described here. This finding can be used to differentiate between agenesis and occlusion of the carotid. In agenesis, there may be narrowing of the common carotid with hypoflow, whereas an occlusion will not have this gradual transition of diameters. 

 Investigation of the presence of the carotid canal in axial plane cranial tomography can also differentiate between these two entities. In agenesis, it will be noted that the carotid canal is absent and, when malformed or absent, this prevents development of the internal carotid. Cases of internal carotid atresia may indicate a hypoplastic carotid canal. 6

 Conventional magnetic resonance may show that the flow void phenomenon is not present. In normal situations, blood at high flow rates through the internal carotid cannot return the radio signal in time and the flow does not appear on the scan. 4

 Digital subtraction angiography may show that the internal carotid disappears immediately after its origin, while the diameter of the external carotid may be larger than normal. Teal et al. consider that angiography demonstrating absence of an internal carotid combined with tomography demonstrating absence of the carotid canal is sufficient to diagnose agenesis. 6

 As explained in this report, it is important to suspect vascular malformations when investigating neurological presentations. Agenesis of the carotid is rare and tends to cause few symptoms, but there is a significant association with cerebral aneurysms. If such aneurysms are identified in time, the patient can be saved from serious complications. Very often, the first clinical findings related to this malformation are nonspecific symptoms such as headaches, syncope, or a transitory ischemic episode or encephalic vascular hemorrhagic. It is important to consider the possibility of carotid agenesis in the investigation when other more prevalent causes have already been ruled out. Diagnosis can be confirmed with imaging examinations that show the absence of an internal carotid, such as angiography, angiotomography, or magnetic resonance, combined with absence of the carotid canal. 

 Additionally, cerebrovascular insufficiency may be precipitated in patients with severe atherosclerotic disease who also have agenesis of an internal carotid. Early diagnosis is therefore very important for optimization of clinical treatment and planning of possible surgical treatment in the future. 
