# Supplementary material for: Agenesis of the internal carotid artery
Source: J Vasc Bras. 2018 Jul-Sep;17(3):243–7. doi: 10.1590/1677-5449.001918 (PMC6326140; doi:10.1590/1677-5449.001918)
Supplement: Translation

# Translation: Agenesia de artéria carótida interna

**Authors:** Adriano Carvalho Guimarães, Thaís Duarte Baião Pessoa, Ricardo Herkenhoff Moreira, Walter Junior Boim de Araujo

**Affiliations:** 1 V&P Health Excelência Médica, Santo Antônio da Platina, PR, Brasil.; 2 Hospital Angelina Caron, Serviço de Cirurgia Vascular, Campina Grande do Sul, PR, Brasil.; 3 Hospital Nossa Senhora da Saúde, Santo Antônio da Platina, PR, Brasil.; 4 Universidade Federal do Paraná (UFPR), Departamento de Cirurgia, Curitiba, PR, Brasil.

**Keywords:** artéria carótida, agenesia, aneurisma cerebral

## Abstract

A agenesia de carótida interna é uma anomalia rara. Na maioria dos casos, é assintomática devido às anastomoses que podem estar presentes, mas pode estar associada a complicações, principalmente quando evidenciada a presença de outras alterações anatômicas ou doença aterosclerótica grave. Relatamos o caso de uma paciente feminina de 63 anos, hipertensa e diabética, com história de cirurgia prévia para clipagem de aneurisma cerebral. Na investigação através de eco-Doppler e angiotomografia de carótidas e vertebrais, foi evidenciada agenesia unilateral da artéria carótida interna esquerda. Este relato objetiva chamar atenção para a importância de se suspeitar de malformações vasculares durante a investigação de quadros neurológicos. A agenesia de carótida interna tem uma importante associação com aneurismas intracerebrais, e é possível poupar o paciente de graves complicações quando estes são identificados a tempo.

## INTRODUÇÃO

 O sistema arterial cervical e cerebral passa por várias transformações ao longo do desenvolvimento embrionário até alcançar o modelo final, no feto. A concepção desse sistema é modulada por inúmeros fatores moleculares, e falhas nessas vias podem causar variações anatômicas e diferentes repercussões clínicas. A aorta primitiva possui seis arcos que se organizam nos diferentes ramos conhecidos, sendo que o terceiro arco origina as carótidas comuns e os segmentos proximais das carótidas internas. Os segmentos distais são derivados da aorta dorsal entre o primeiro e o terceiro arco primitivo. As carótidas externas surgem das carótidas comuns. Em aproximadamente 65% da população ocorre esse padrão de desenvolvimento; nos demais, anomalias podem ser observadas 1 . 

 As anomalias resultam da persistência ou do desaparecimento indevido de segmentos do arco da aorta primitiva. Em 22% da população, a artéria carótida comum esquerda é originária do tronco braquiocefálico em vez de se originar do arco aórtico, também denominado “arco bovino”. Nesse caso, o tronco braquiocefálico dá origem à artéria subclávia direita, bem como às artérias carótidas comum direita e esquerda, enquanto a artéria subclávia esquerda é originária do arco aórtico, como normalmente esperado. Essa variante representa 73% de todas as anomalias do arco. Muitas outras variações, cada uma ocorrendo em menos de 3% da população, foram descritas. 

 A agenesia de carótida interna foi descrita pela primeira vez em 1787 *post-mortem* e, em 1954, pela primeira vez *in vivo* após exame de angiografia 2 . É uma anomalia rara com incidência menor que 0,01% 3 , sendo, na maioria dos casos, assintomática devido às anastomoses que podem estar presentes. No entanto, pode estar associada a complicações, principalmente quando evidenciada a presença de outras alterações anatômicas ou doença aterosclerótica grave. 

## RELATO DO CASO

 Paciente de 63 anos, sexo feminino, era hipertensa e diabética. Não tinha história de tabagismo e doença cardíaca. Apresentava histórico de cirurgia prévia para clipagem de aneurisma cerebral havia 3 anos e relato na ocasião, pelo médico neurocirurgião responsável, de dificuldades para o cateterismo das artérias cervicais. Foi então submetida a eco-Doppler de carótidas e vertebrais, que evidenciou artéria carótida comum esquerda com menor calibre quando comparada à direita ( Figura 1 ), impossibilidade de visualização da bifurcação carotídea esquerda e continuação da artéria carótida comum esquerda apenas com a artéria carótida externa esquerda ( Figura 2 ). Foi então solicitada uma angiotomografia, que evidenciou agenesia de artéria carótida interna esquerda ( Figura 3 ). A paciente se encontra assintomática e em acompanhamento regular. 

**Figura 1:** Imagens ecográficas em modo B evidenciando artéria carótida comum esquerda (ACC ESQ) com menor calibre quando comparada à artéria carótida comum direita (ACC D).

**Figura 2:** Imagens de eco-Doppler evidenciando perviedade da artéria carótida comum esquerda (ACC ESQ) (A) e continuidade com a artéria carótida externa esquerda (ACE ESQ) (B).

**Figura 3:** Angiotomografia evidenciando perviedade da artéria carótida comum esquerda (ACC ESQ) (seta branca) em continuidade com a artéria carótida externa esquerda (ACE ESQ) (seta amarela) e agenesia de artéria carótida interna esquerda (ACI ESQ).

## DISCUSSÃO

 A agenesia de carótida interna geralmente é unilateral. Nesses casos, o principal suprimento sanguíneo compensatório é a carótida interna contralateral. Nos casos de agenesia bilateral, o sistema vertebrobasilar pode assumir essa função. A maioria dos casos não apresenta sintomas, fato que pode ser atribuído à abundante rede anastomótica, incluindo o polígono de Willis, vasos intracavernosos e ramos da carótida externa, bem como artérias embriológicas persistentes. Já foram relatadas discreta predominância em homens e predileção à esquerda em uma taxa de 3:1 4 ^-^ 6 . 

 Segundo Lie, o termo agenesia significa ausência completa de órgão ou estrutura, enquanto aplasia é decorrente de um lapso no desenvolvimento do órgão – sendo que o seu precursor existe – e hipoplasia é atribuída ao desenvolvimento incompleto. Acredita-se que a agenesia unilateral se deva a um estresse intrauterino mecânico ou hemodinâmico. A principal hipótese é que ocorra uma rotação exagerada do embrião em direção a um lado ou uma constrição por brida amniótica. Já a agenesia bilateral ainda tem causa desconhecida 5 ^,^ 7 . 

 O desenvolvimento da carótida interna se inicia no estágio no qual o embrião apresenta o *crown-rump* (CR, a distância entre o vértice do crânio até o ponto médio entre os ápices das nádegas) de 4-5 mm e se completa na 6ª semana de gestação (CR =10-14 mm). O polígono de Willis se forma no estágio de 7-24 mm (cerca de 7 semanas de idade gestacional). O padrão de fluxo sanguíneo colateral, se houve má-formação de carótida, e a vasculatura intracraniana dependem do estágio em que ocorreu a interrupção no desenvolvimento da artéria. Cali et al. postularam que a circulação colateral prevalece pelas vias primitivas (anastomoses intracavernosas) caso a interrupção no desenvolvimento da carótida interna ocorra antes da conclusão do polígono de Willis. Se a interrupção se dá após sua conclusão, a circulação colateral prevalece pelas anastomoses intracavernosas. Poucos casos de agenesia de carótida interna têm sido relatados em crianças, o que sugere que inicialmente as colaterais são capazes de compensar o fluxo. Os casos sintomáticos são, na sua maioria, identificados na fase adulta, na qual fatores como a aterosclerose podem precipitar a insuficiência cerebrovascular 2 ^,^ 8 . 

 Lie descreveu seis padrões de circulação colateral associada à ausência de carótida interna. No tipo A, tem-se a ausência unilateral da carótida interna associada a uma circulação colateral com a artéria cerebral anterior (ACA) ipsilateral através da artéria comunicante anterior (ACOM) e com a artéria cerebral média (ACM) ipsilateral através da artéria comunicante posterior (PCOM), usualmente hipertrofiada. No tipo B, há ausência unilateral de carótida interna associada a colaterais com ACA e ACM através da ACOM. No tipo C, tem-se uma agenesia bilateral, e a circulação anterior é suprida pelo sistema vertebrobasilar através de uma PCOM hipertrofiada. O tipo D se caracteriza por agenesia unilateral da porção cervical da carótida interna associada a colaterais intracavernosas com o segmento intracavernoso da carótida interna contralateral. No tipo E, há hipoplasia bilateral de carótidas internas, e suas ACA e ACM são supridas por PCOMs hipertrofiadas. No tipo F, tem-se ausência bilateral sendo compensada por anastomoses transcraniais e ramos da carótida através da *rete mirabile* 7 . Desde a descrição de Lie em 1968, outros autores têm reportado a presença de uma artéria trigeminal persistente envolvida em colaterais para reconstituição do fluxo na agenesia de carótida interna unilateral 5 . É importante a identificação de anastomoses compensatórias – como as intracavernosas – para que não ocorra complicações peroperatórias devastadoras como em cirurgias transesfenoidais para acesso à hipófise. 

 Outras anomalias, como aneurismas intracranianos, podem acompanhar cerca de 24-67% dos casos de agenesia e se associar a hemorragia intracraniana, enquanto na população normal aneurismas são encontrados em 2-4%. Cerca de 25% dos casos sintomáticos de agenesia podem cursar com aneurismas e sangramentos destes. A causa dessa prevalência mais elevada pode residir no fato de haver um aumento de fluxo nas vias colaterais, o que eleva o estresse parietal nesses vasos. A ACA tem sido relatada como a colateral mais acometida por aneurismas nessa situação. Alguns autores descrevem o sangramento subaracnóideo decorrente de aneurismas como achado inicial da agenesia de carótida interna 6 . No caso relatado, havia aneurisma associado, que foi tratado antes de ocasionar complicações. Alguns pacientes podem apresentar também atraso do desenvolvimento neuropsicomotor, cefaleias e episódios de ataque isquêmico transitório 3 ^,^ 4 . 

 Por ser, na maioria das vezes, assintomática, essa anomalia geralmente é diagnosticada incidentalmente em exames de imagem. Quando isso ocorre, é importante descartar se o hipofluxo identificado tem causa em uma estenose adquirida ou anomalia congênita do vaso ou canal ósseo 3 ^,^ 4 . Essa diferenciação tem sua importância no fato de que a agenesia pode estar associada a outras anomalias, como aneurismas, que devem ser pesquisadas antes do surgimento de complicações. 

 A anomalia pode ser identificada em exame de ultrassonografia com Doppler, no qual não se consegue visualizar a bifurcação carotídea ou se percebe uma hipoplasia da carótida comum ipsilateral, como visto no caso relatado. Esse achado pode fazer a diferenciação entre um quadro de agenesia ou de oclusão de carótida. Na agenesia, pode-se notar um afilamento da carótida comum com hipofluxo, ao passo que na oclusão não existe essa transição gradual de diâmetros. 

 A investigação da presença de canal carotídeo através de tomografia de crânio em planos axiais também pode diferenciar essas duas entidades. Na agenesia, nota-se ausência de canal ósseo carotídeo, que, quando malformado ou ausente, inviabiliza o desenvolvimento da carótida interna. Casos de atresia de carótida interna podem refletir um canal carotídeo hipoplásico 6 . 

 Exames de ressonância magnética convencionais podem mostrar a ausência do fenômeno do *flow void*. Em situações normais, pelo alto fluxo na carótida interna, o sangue não consegue devolver o sinal de rádio a tempo, ficando “vazia de fluxo” no exame 4 . 

 Na angiografia digital por subtração, pode-se perceber a ausência da carótida interna imediatamente após sua origem, ao passo que a carótida externa pode ter maior diâmetro que o comum. Teal et al. acreditam que uma angiografia demonstrando ausência de carótida interna associada a uma tomografia demonstrando ausência do canal carotídeo é suficiente para o diagnóstico da agenesia 6 . 

 Conforme evidenciado ao longo deste relato, é importante suspeitar de malformações vasculares durante a investigação de quadros neurológicos. A agenesia de carótida é rara e tende a ser pouco sintomática, porém há importante associação com aneurismas intracerebrais. Quando os aneurismas são identificados a tempo, pode-se poupar o paciente de graves complicações. Muitas vezes, os primeiros achados clínicos dessa má-formação se traduzem em sintomas inespecíficos como cefaleias, síncopes ou no próprio ataque isquêmico transitório ou acidente vascular encefálico hemorrágico. É importante considerar a possibilidade de uma agenesia de carótida na investigação quando outras causas mais prevalentes já foram descartadas. O diagnóstico pode ser confirmado com exames de imagens que demonstrem a ausência da carótida interna, como angiografia, angiotomografia ou ressonância magnética, associada à ausência do canal carotídeo. 

 Além disso, pacientes portadores de doença aterosclerótica grave podem ter insuficiência cerebrovascular precipitada se forem portadores da agenesia de carótida interna. O diagnóstico precoce é, portanto, de suma importância para otimização do tratamento clínico e planejamento de possível tratamento cirúrgico no futuro.
